# Behçet’s disease in children, an overview

**DOI:** 10.1186/s12969-016-0070-z

**Published:** 2016-02-18

**Authors:** Isabelle Koné-Paut

**Affiliations:** Paediatric Rheumatology, CEREMAI, Bicêtre University Hospital, APHP, Paris SUD, Kremlin bicêtre, France

**Keywords:** Behçet’s disease, Children, Clinical features, Epidemiology, Classification, Treatment

## Abstract

BD is a systemic inflammatory disease with a variable vasculitis. Paediatric onset is very rare and carries a strong genetic component. Oral ulcers and fever of unknown origin are frequent at onset and difficult to distinguish from other inflammatory disorders; therefore, expert opinion is still mandatory to recognize the disease early. An international expert consensus has recently proposed new classification criteria for children with BD. The clinical spectrum of BD is heterogeneous and influenced by gender, ethnicity and country of residence. Young males have the worst prognosis with significantly more frequent neurological, ocular and vascular involvement. BD treatment is aimed at alleviating inflammation. Among all drugs, TNFα inhibitors have become a standard to control severe ocular, neurological and digestive system involvement.

## Background

Behçet’s disease (BD) is a systemic inflammatory disease involving primarily the oral and genital mucosa, skin and eyes. BD includes a vasculitis affecting all sizes of vessels but prominently the veins; thus, it was recently reclassified as variable type, [1, 2]. The onset is insidious with peak age of onset in young adulthood (25–30 years), but also occasionally in children before the age of 16 years in 4 to 26 % of cases [3, 4]. As observed in autoinflammatory diseases, BD attacks are recurrent and self-limited. Neurological and ocular involvement cause substantial functional limitation and handicaps. Large vessel vasculitis is the leading cause of death, typically by multiple thrombosis or pulmonary arteritis. Even though it is considered as a single entity, the clinical presentation of BD is heterogeneous and may vary according to sex, country of residence and age of onset [5–9]. The underlying mechanisms causing the disease are still unclear and could combine both autoinflammation and autoimmunity. The recent discovery of A20 protein haploinsufficiency as a cause of autosomal dominant “Behçet-like” disease strongly supports the idea that the full spectrum of disorders meeting criteria for BD cannot be related to a single mechanism [10].

## Definition- classification of BD

The definition of BD is difficult and relies only on clinical features. In addition, BD shares considerable overlap of clinical features with other autoinflammatory disorders, spondyloarthritis, and some immunodeficiencies. The International Study Group for BD proposed the most widely used classification in 1990: recurrent oral ulceration (OU) at least thrice a year, as mandatory criteria, associated with at least two other criteria among a list of cutaneous and ocular symptoms, that gave a diagnostic sensitivity of 85 % and a specificity of 96 % (Tables 1, 2 and 3) [11]. The relatively low sensitivity of this classification was related to the load given to OU, which can be absent in 5 % of patients, and to the positivity of the pathergy test, which is not applicable in all populations. In 2014 an International Team for the Revision of the International Criteria for BD (from 27 countries) proposed new criteria based on 2556 clinically diagnosed BD patients and 1163 controls. The main changes from the previous classification are that OU and pathergy test are not mandatory. In addition, vascular and neurological symptoms are listed.. A score of 4 points or more is needed for diagnosis (Tables 1, 2 and 3) [12]. Our group has recently published provisional classification criteria from the largest prospective cohort ever reported for BD in children [13]. All the clinical symptoms have the same weight and the pathergy test is not included (Tables 1, 2 and 3). This classification has been proposed for the main purpose of clinical research; indeed, in children the number of symptoms may be too few to apply any classification to a single patient. Finally, in most cases the diagnosis is made provisionally on the basis of the physician’s expertise.

**Table 1:** International classifications criteria for Behçet’s disease [11–13] - Criteria of the international study group for BD

Recurrent oral aphtosis (mandatory, seen by a physician and recurrence at least 3 times in a year)	
**plus at least two of **	
Genital ulceration *(or aphthosis, at least once)*	
Skin lesions *(erythema nodosum, necrotic folliculitis and papulopustular lesions, (post adolescent, not receiving steroids)*	
Ocular lesions *(posterior uveitis, total uveitis, retinal vasculitis)*	
Positive pathergy test *(done by needle prick (oblique insertion of 20- gauge needle) or intradermal saline injection and read at 48h by a physician. Positive reactions include: papula, pustula or papula with surrounding erythema)*

**Table 2:** International classifications criteria for Behçet’s disease [11–13] - International criteria 2014

Clinical manifestations	Description	Value/item
Oral aphthosis		2
Genital ulceration		2
Ocular signs	Anterior uveitis, posterior uveitis, retinal vasculitis	2
Skin lesions	Pseudo folliculitis, skin aphthosis, erythema nodosum	1
Neurological manifestation		1
Vascular signs	Arterial thrombosis, Large vein thrombosis, phlebitis or superficial phlebitis	1
*Positive pathergy test*	*(additional criteria)*	1

Behçet’s disease diagnosis is made if the score is ≥ 4

**Table 3:** International classifications criteria for Behçet’s disease [11–13] - Paediatric criteria for BD 2015

Item	Description	Value/item
Recurrent oral aphtosis	At least 3 attacks/year	1
Genital ulceration	Typically with scar	1
Skin involvement	Necrotic folliculitis, acneiform lesions, erythema nodosum	1
Ocular involvement	Anterior or posterior uveitis, retinal vasculitis	1
Neurological signs	With the exception of isolated headaches	1
Vascular signs	Venous thrombosis, arterial thrombosis, arterial aneurysm	1

Three of 6 items are required to classify a patient as having paediatric BD

## Etiology/pathogenesis

The aetiology of BD is still unknown; however, it appears to be a complex disease relying on an interaction between the genetic background and the environment. The role of innate immunity through infectious mechanisms has been suggested since the initial description of the disease by Huluci Behçet. Pathogens such as herpes simplex virus, streptococci, staphylococci, or *Escherichia* species, and heat- shock proteins may contribute to attacks of BD in susceptible persons via stimulation of the innate immune system (inflammasome activation) through TLR2 and TLR4 [14–16], In addition, because OU is often the presenting symptom of BD, the composition of the oral mucosa and salivary microbiome is thought to be one trigger of disease flares [17]. Improvement of dental and periodontal hygiene has been associated with a decrease of oral ulcerations, and could be one factor explaining the decrease of the incidence of BD in countries like Japan and Turkey [18].

## Immunology

A major feature of BD is the hyperactivity of neutrophils, an effector cell of the innate immune system, as demonstrated clinically by the pathergy phenomenon following minimal trauma and needle prick, and histologically in affected tissues. However, much is unknown about the immunologic mechanisms contributing to pathogenesis. Since neutrophil activation is influenced by monocytes, NK cells, and T helper (T_H_) -17 cells, studies of these effector cells in BD should yield important insights. IL-21 dependent upregulation of T_H_-1 cells, increased numbers of T_H_-17 cells, increased IL-17A secretion, and decreased numbers of regulatory T cells (Tregs), have all been demonstrated in blood and tissues of BD patients, suggesting an important role for IL-21 in pathogenesis [19]. A therapeutic effect of Interferon-α may be explained by its induction of memory T-cells from BD patients to increase secretion of IL-10, which promoted Treg responses [20]. Although not all studies have demonstrated increased levels of pro-inflammatory cytokines in active BD, inflammasome assembly and activation in monocytes following TLR binding would induce secretion of IL-1β, with secondary induction of IL-6 and TNFα. The efficacy of inhibitors of these cytokines for some manifestations of Behcet’s disease support their pathogenic role (see Treatment).

## Genetics

BD carries a strong genetic component. The frequency of familial cases is reported to be 10 to 50 %, depending of the country and age of onset [21, 22]. HLA-B5, and more specifically its predominant suballele HLA-B51, are associated with BD; carriage predominates in affected males and is associated with a moderately higher prevalence of genital ulcers, ocular and skin manifestations, and a decreased prevalence of gastrointestinal involvement [23, 24]. Besides HLA-B51, which remains its strongest genetic risk factor, other HLA class I alleles such A26, B15, B27, and B56 represent independent risk factors for BD whereas others like A03 and B49 are protective [25]. Genome wide association and next generation sequencing analyses have demonstrated the combined role of genes from both innate and adaptive immunity. Among them, susceptibility loci in ERAP1, IL23R and IL10 are shared with inflammatory bowel disease, psoriasis and spondyloarthritis [26]. In addition, BD association with TLR4, NOD2 and MEFV suggest bacterial and possible other danger sensing mechanisms in BD pathogenesis [27]. An epigenome-wide study of DNA methylation has provided evidence that epigenetic modification of cytoskeletal dynamics underlies BD pathogenesis [28]. Reinforcing the diversity of the disease mechanisms, a novel autoinflammatory disease with a BD-like phenotype (recurrent OU and GU, eye inflammation and axillary abscesses) has been reported in association with haploinsufficiency of A20 protein, a regulator of NFKB activation [10].

## Epidemiology

The geographic distribution of BD is spread along the former Silk Road from the Far East to the Mediterranean basin. As a result, the highest prevalence has been reported in Northern China and Iran (100 per 100,000), Turkey (80–370.0 per 100,000) and the Turkish German population (77 per 100,000). In contrast, BD occurs more rarely in Western Europe with a prevalence of 0.1 per 100,000 in Sweden, 7.1 per 100,000 in France and 15.9 per 100,000 in Southern Italy [29–34]. Besides the country of residence, ethnic background is important, and an epidemiological study performed in the Paris area has shown that the prevalence of BD in immigrants of North African or Asian ancestry is significantly higher than that in the European-origin population [32]. The same phenomenon was observed in Germany [34].

The usual age at BD onset is around 30 years; however, there are numerous observations and case series of paediatric cases. The prevalence of BD in children is unknown but is probably very low, as a range of 3.3 to 26 % of cases has been reported [30, 35]. The international PEDBD cohort study of 156 patients has shown a mean (±SD) age of BD onset (at first symptom) of 7.83 (±4.39) years, in accordance with previous series [6, 7, 35, 37]. Paediatric patients have generally few symptoms, and the time to diagnosis is long - between 3 and 5 years [4, 6, 7, 36]. Familial aggregation is high in paediatric BD, and has been observed in 9 to 42 % of cases [13, 21, 22, 37]. Previous studies had reported variations regarding the sex ratio of BD, with female predominance in Asian populations and male predominance in Middle Eastern and Mediterranean countries. The most recent epidemiological studies tend toward an equal sex ratio, as currently observed in paediatric BD, but with significant phenotypic differences according to gender. Indeed, severe uveitis, vascular disease and mortality occur more frequently in males whereas genital aphthosis and erythema nodosum are more frequent in females [6, 7, 38–40].

## Clinical symptoms

### Fever

Recurrent fevers are scarcely documented (22 %) in the adult BD population where they correlate with vascular, neurological or joint involvement [41]. Recurrent fevers were not significantly associated with the diagnosis of BD in the PEDBD cohort, but they were present in 44 % (68/156) of patients. At this age, if fever remains a calling symptom of severe vascular and neurological disease, it is also observed in association with attacks of oral aphthosis in a clinical presentation resembling Periodic Fever, Aphthosis, Pharyngitis, and Adenitis (PFAPA) syndrome [42].

### Mucocutaneous lesions

Recurrent OU is not exceptional in the general paediatric population, but remains a major clue for the diagnosis of BD. ROU is the presenting symptom in 87 to 98 % of cases and is present in almost all patients with BD (95 %) (Table 4). BD-related OU do not differ from other OU. They are discrete, round or oval ulcers with a yellow-gray pseudomembranous base and a brightly erythematous halo (aphthae); they involve the lips, tongue, cheeks, and palate and disappear without scar. All variations are possible: single, multiple, herpetiform and necrotic, from minor to major. Lesions are painful and may interfere with eating, speaking, or swallowing. Minor lesions heal between 7 and 10 days but major lesions may evolve over weeks. When they are isolated, a large differential diagnosis is necessary to rule out numerous other causes of ROU, including autoinflammatory diseases, inflammatory bowel diseases (IBD) and immunodeficiency. OU may be the only manifestation of disease for an average of 6 to 7 years before the second major manifestation is apparent [43, 44]. GU is observed in 55 to 83 % of children with BD, which is less than in the adult population (80 to 90 %). In the PEDBD study they appeared at a mean (±SD) age of 11.23 ± 4.32 years [13]. The lesions are usually larger and deeper than OA and have an irregular margin [15]. They are located typically on the vulva and the scrotum; in children, skin aphthae over the perianal region has been observed in 7 % of patients [7], and must be distinguished from anal aphthosis which is more related to IBD. In children, the combination of OU plus GU is not sufficient to confirm the diagnosis of BD. (Tables 1, 2 and 3). Other cutaneous symptoms are very frequent in BD; in the PEDBD cohort they appeared later than OU, at a mean age of 10.36 (±4.30) years. Symptoms associated with PEDBD classification were necrotic folliculitis, and acneiform lesions more commonly in males, and erythema nodosum more commonly in females [6]. These lesions appear often in combination and primarily involve the buttocks and the lower limbs. Other neutrophilic rashes may also be observed as well as lesions corresponding to superficial thrombosis (dermal nodules). The skin of patients with BD is irritable at puncture, minor trauma and shaving. The pathergy test, using a 24 gauge needle puncture, was formerly used to demonstrate skin hypersensitivity (pustula in 24 to 48 h) [11]. However the positivity of this test varies from 40 to 80 % according to the studied population. The pathergy test is no longer mandatory to define BD in adults, and is not retained as a criterion for BD in children [13].

**Table 4:** Frequencies of clinical characteristics of BD in children compared to adults

	Paediatric series					Adult series	
Clinical features in %	Karincaoglu [4]	Krause [37]	Koné- Paut [7]	Atmaca [35]	Koné-Paut + [13]	Alpsoy E [38]	Krause [37]
*N* = patients	33	19	65	110	156	661	34
Oral aphthosis	100	100	96	100	100	100	100
Genital aphthosis	82	31.6	70	83	55	58.3	88.2
Cutaneous signs		89.5	92	76			82.4
Erythema nodosum	52	36.8	40	37	22.7	44.2	26.5
Pseudo folliculitis	51	8/19	58	39	43	55.4	ND
Pathergy test +	37	41.2	-	45.5	NS	37.8	57.1
Ocular signs	35^b^		60		45	29.2	*Approx* 50 %
Anterior uveitis		47.4		31	30.1		47.1
Posterior uveitis		42.1	36	9 %	28		8.8
Retinal vasculitis		10.5			16.7		
Arthralgia/arthritis	40	47.4	56	22	41	33.4	17.6
Gastro intestinal	4.8	36.8	14	NS	4.49	1.6	11.7
Neurological^a^	7.2^a^	26.3	15	3.6^a^	5^a^	3^a^	5.8^a^
Vascular	9.6	10.5	15	3.6	15.38	4.4	26.5
Family history of BD	19	ND	15	12	24.4	ND	ND
Age first symptom (years)	12.3 (1–16)	6.9 ± 3.9	8.4 (0–16)	11.6 (1–16)	7.83 ± 4.39	ND	30.4 ± 8

^a^Other than headaches ^b^uveitis without details + in this study patienst were classified by an international expert committee, independently of the international criteria

### Joint involvement

Oligo and polyarthritis develop in half of adult patients [45]. Twenty to forty percent of children with BD have some articular involvement, generally arthralgia in few joints (knees, ankles, elbows and wrists). Joint symptoms were present in 50 % (78/156) of definite BD patients in the PEDBD study. Axial arthritis was reported in 16.67 % (26/156, which is rather high), and peripheral arthritis in 47.44 % (74/156) [13]. The course is recurrent. Association with HLA- B27 spondylarthropathy is seen in 2 % of patients.

### Eye involvement

Bilateral posterior uveitis with retinal vasculitis is the most typical feature of ocular-BD. In this case, patients usually have a painless, bilateral decrease in visual acuity [15]. The mean (±SD) age of onset of uveitis was 10.94 ± 3.62 years in the PEDBD cohort [13]. Anterior uveitis could be more frequent before the age of ten years in association with familial aggregation but panuveitis could start later. Intermediate uveitis is rare. Uveitis may be the presenting symptom of BD in 10 % of cases, and it has been recognised that patients with uveitis suffer generally less of OU. In a Tunisian retrospective survey of 62 patients (111 eyes), panuveitis (68 eyes, 61.3 %) and posterior uveitis (38 eyes, 34.2 %) were the most common forms, followed by anterior uveitis (five eyes, 4.5 %). Retinal vasculitis was found in 89 eyes (80.2 %) [46]. The most common complications included posterior synechiae (32.4 %), cataract (31.5 %), and cystoid macular edema (19.8 %). Similar findings were observed in adult and childhood uveitis from several case series [47-49]. In the PEDBD registry at a mean of 5 years of follow up, visual acuity was decreased to <1/10 (equivalent to <20/200 by Snellen test) in 16.6 % (26/156) of patients, which represented 33.3 % (26/78) of patients with ocular involvement [13]. The occurrence and severity of uveitis is associated with the male sex, generally after the age of ten years [48, 49].

### Neurological involvement

Neurologic involvement is newly considered in the definition of BD and occurs in 5.3 to 59 % of adult cases [50]. Chronic headaches are frequent in patients with BD, and may be either isolated or associated with severe NBD involvement [51]. In the PEDBD study, although not considered as a classification criterion, the presence of headaches was significantly associated with BD confirmation by the experts (*p* = 0.0063) [13]. NBD manifestations include acute and chronic progressive manifestations, especially in young males with early onset of disease. Acute manifestations include recurrent aseptic meningitis and meningoencephalitis. The CSF examination shows elevated protein levels, and pleocytosis consisting of both polymorphonuclear cells and lymphocytes [15]. Acute manifestations are generally relapsing but responsive to steroids. Parenchymal lesions are located in the brainstem, and multiple high-intensity focal lesions in the brainstem, basal ganglia, and cerebral white matter are typical findings on T2-weighted MRI [15, 50, 52, 53]. Patients present with acute headaches, bilateral papilledema and hemiparesis accompanied with signs of brainstem involvement (ataxia, pyramidal and extrapyramidal syndrome, epilepsy, and cranial nerve involvement). Chronic progressive lesions are parenchymal and vascular. They manifest as various neuropsychiatric disturbances such as cognitive impairment, memory loss, depression, anxiety, pseudobulbar syndrome and definite sensitive and motor deficits. These impairments are generally irreversible [15, 50]. Other lesions not classified as parenchymal or vascular are seldom observed, like myositis (myopathy) and peripheral neuropathy. The frequency of NBD in children ranges from 15 to 30 % and increases up to 50 % considering isolated headaches which can be the presenting symptom of BD in up to 25 % of reported cases [13, 53]. The main manifestations at the paediatric age are cerebral venous thrombosis, and cranial nerve palsy (especially the VI ^th^ nerve), rather than parenchymal lesions. The rate of neurologic sequelae reached (75 %) in one study, and had long-term consequences on children’s schooling. Half of the NBD children could not follow a normal curriculum and 50 % of the remainder needed extra school arrangements to be maintained in normal school system [53].

### Vascular involvement

BD affects any type and size of vessel, and was recently classified as “variable” vasculitis [1]. The vascular involvement is now integrated in both the adult and the paediatric BD classification criteria. The main pathologic feature is an inflammation of the vessel wall leading to thrombus formation. Activated neutrophils produce excessive superoxide anion radicals and lysosomal enzymes that induce lesions of the vessel wall. Endothelial damage with aneurysm formation causes local blood flow abnormalities. The thrombus is usually adherent and does not generate secondary embolism. In a French retrospective cohort study of 807 BD patients 36.7 % (296 patients, 73.3 % male, median age 30 years) had venous thrombosis. Deep vein thrombosis of the legs was the most frequent vascular event (55 %); 10.9 % had vena cava involvement, 9.7 % pulmonary artery involvement, 2.2 % cervical vein thrombosis, and 2.4 % Budd-Chiari syndrome [54]. At 5 years the mortality rate was 6.4 % without immunosuppressive treatment. The frequency of relapse is high; in a Turkish study, first vascular relapse developed in 32.9 % of patients and the time interval between the first and second vascular event was 25.5 (1–252) months [55]. In children, if the condition per se is a risk for vascular activation, male patients are predominantly affected (SR 6/1) and many patients may accumulate other factors of thrombophilia such as anticardiolipin antibodies and protein C deficiency [56]. The frequency of vascular involvement is reported to be 5 to 20 % in paediatric series of BD (Table 4). In a series of 47 adults with pulmonary artery vasculitis, the presentation was usually haemoptysis (79 % of cases) associated with cough, fever, dyspnea, and pleuritic chest pain. The major risks were aneurysm and/or thrombus formation with secondary rupture or embolism, which generally evolved in patients with active extra-pulmonary disease. After a mean follow-up of 7 years, 12 of 47 (26 %) patients were dead, especially those with the larger aneurysms [57].

### Digestive involvement

Gastrointestinal (GI) involvement of BD is a challenging situation, which raises the possibility of another IBD. In children, GI symptoms occurred in 40.38 % (61/156) of patients in the PEDBD study, and were essentially isolated abdominal pain or discomfort. Other symptoms were digestive aphthae 4.49 % (7/156), bleeding 2.56 % (4/156), and perforation (one case) [13]. The most severe cases of GIBD are reported from Japan and Korea with a frequency of 40 %. The clinical symptoms of colitis have no specificity: bloody diarrhoea, nausea, fever, weight loss. Lesions may involve the whole digestive tract from the mouth to the anus. The ileum and the colon are the most commonly involved intestinal sites. Deep aphthous and necrotic ulcerations lead to abscesses and perforation requiring surgery.

### Miscellaneous involvement

Pulmonary involvement affects primarily the pulmonary arteries, usually at multiple sites, but parenchymal lesions such as nodules and cavities are reported. Pleural effusions and mediastinal lymphadenopathy are also part of BD; however, ruling out infectious causes is mandatory [57]. BD vasculitis involves the aorta, the sinuses of Valsalva, and the coronary arteries [58]. Pericarditis is common, variably manifesting as recurrent, hemorrhagic, or small asymptomatic pericardial effusion. Myocarditis and intracardiac thrombus are also seen occasionally. BD-associated diseases include several types of glomerulonephritis, crescentic glomerulonephritis and IgA nephropathy being the most common [59]. Tubulointerstitial nephropathy is rare. AA amyloidosis has decreased with time and better management of BD. Orchitis, epididymitis, urethritis and sterile cystitis are rare.

## Disease course and prognosis

The course of BD is recurrent and unpredictable. In children the disease remains often active with new symptoms appearing with time. Functional impairments are related to neurological and ocular disease. In adults BD symptoms usually decrease with time after a mean follow up of ten years. In a single-center retrospective cohort of 817 children and adults followed for a median of 7.7 years, the mortality rate was 5 %. Vascular disease was the main cause of death, (43.9 % of deaths), followed by malignancy (14.6 %), CNS involvement, (12.2 %) and sepsis (12.2 %). Death was associated with younger age (15–25 years), male sex, arterial involvement, and a high number of flares [39].

## Value of biological exams

The acute phase reactants, i.e. CRP and ESR, may not be modified, except in case of active serositis, cerebral involvement or vasculitis. BD cannot be ruled out in the absence of the antigen HLA-B51.

## Treatment

The treatment of BD is aimed at relieving inflammation. The treatment of BD in children follows the 2009 international recommendations [60]; of interest, none of these treatments have been currently approved by the FDA or EMA for the indication of BD. Colchicine is widely used to control recurrent oral and genital ulceration; however, no studies have provided evidence to demonstrate its efficacy. Short courses of systemic steroids may help alleviate severe mucous ulcerations but cannot be used on a long-term basis. Other therapeutic options for chronic lesions include topical steroids and analgesics, oral azathioprine, and TNFα inhibitor treatments. Oral thalidomide is an effective treatment but its use is limited by its frequent neurological toxicity [61]. In a recent phase II placebo-controlled study of 111 BD patients with recurrent OU, Apremilast (Otezla®), an oral phosphodiesterase-4 inhibitor, reduced significantly the mean (±SD) number of oral ulcers per patient at week 12, compared to placebo (0.5 ± 1.0 vs. 2.1 ± 2.6) (*P* < 0.001) [62]. Severe vascular, neurologic or ocular involvement requires a combination of steroids and immunosuppressive treatment (azathioprine, cyclosporine, cyclophosphamide). Among them, azathioprine is still widely used as steroid sparing treatment. TNF-α inhibitors have become standard treatment of severe neurological, digestive and ocular involvement for patients resistant to conventional immunosuppressants [63]. Several reports have mentioned the use of anti IL1 (anakinra, canakinumab) and anti IL6 blockers (tocilizumab) with various results in treating selective manifestations of BD, and two exploratory phase II studies have shown efficacy and safety of Gevokizumab in patients with steroid dependant uveitis [64–67]. The use of anticoagulants in treating BD thromboses is still controversial and no studies have demonstrated their efficacy in comparison to immunosuppressive treatment [68].

## Conclusions

BD is still a difficult disease in terms of both diagnosis and treatment. The new paediatric classification criteria may increase the opportunity of therapeutic trials in paediatric patients, however they need further validation. The use of anti TNFa treatment has become a standard for severe ocular, neurological and digestive involvement.
